# Identification and Mendelian randomization validation of pathogenic gene biomarkers in obstructive sleep apnea

**DOI:** 10.3389/fneur.2024.1442835

**Published:** 2024-08-16

**Authors:** Nianjin Gong, Yu Tuo, Peijun Liu

**Affiliations:** ^1^Department of Respiratory and Critical Care Medicine, The Central Hospital of Enshi Tujia and Miao Autonomous Prefecture, Enshi, China; ^2^Department of Oncology, The Central Hospital of Enshi Tujia and Miao Autonomous Prefecture, Enshi, China

**Keywords:** obstructive sleep apnea, differentially expressed genes, WGCNA, Mendelian randomization, immune infiltration

## Abstract

**Background:**

By 2020, obstructive sleep apnea (OSA), a prevalent respiratory disorder, had affected 26.6–43.2% of males and 8.7–27.8% of females worldwide. OSA is associated with conditions such as hypertension, diabetes, and tumor progression; however, the precise underlying pathways remain elusive. This study aims to identify genetic markers and molecular mechanisms of OSA to improve understanding and treatment strategies.

**Methods:**

The GSE135917 dataset related to OSA was obtained from the GEO database. Differentially expressed genes (DEGs) were subsequently identified. Weighted gene co-expression network analysis (WGCNA) was conducted to pinpoint disease-associated genes. The intersection of these data enabled the identification of potential diagnostic DEGs. Further analyses included Gene Ontology and Kyoto Encyclopedia of Genes and Genomes enrichment studies, exploration of protein–protein interactions based on these genes, and an examination of immune infiltration. Mendelian randomization was employed to validate core genes against the Genome-Wide Association Study database.

**Results:**

A total of 194 DEGs were identified in this study. WGCNA network analysis highlighted 2,502 DEGs associated with OSA. By intersecting these datasets, 53 diagnostic DEGs primarily involved in metabolic pathways were identified. Significant alterations were observed in immune cell populations, including memory B cells, plasma cells, naive CD4 T cells, M0 macrophages, and activated dendritic cells. CETN3, EEF1E1, PMM2, GTF2A2, and RRM2 emerged as hub genes implicated in the pathogenesis. A line graph model provides diagnostic insights. Mendelian randomization analysis confirmed a causal link between CETN3 and GTF2A2 with OSA.

**Conclusion:**

Through WGCNA, this analysis uncovered significant genetic foundations of OSA, identifying 2,502 DEGs and 194 genes associated with the disorder. Among these, CETN3 and GTF2A2 were found to have causal relationships with OSA.

## Introduction

Obstructive sleep apnea (OSA) is a prevalent chronic sleep disorder affecting individuals globally. In the United States, approximately 10% of adults experience mild OSA, with moderate to severe cases ranging from 3.8 to 6.5% (1, 2). The primary symptom of OSA is repetitive upper airway collapse during sleep, largely due to the activity of the genioglossus muscle. This collapse leads to sleep disruptions and intermittent hypoxia, causing daytime fatigue and drowsiness. Moreover, OSA significantly increases the risk of various conditions, including coronary heart disease, diabetes, and cerebrovascular accidents, creating a substantial health and economic burden on individuals and society (3). Polysomnography is the primary diagnostic tool for OSA (4). However, its limited availability in specialized medical institutions and issues with patient discomfort challenge its widespread use (5). OSA often results in chronic intermittent hypoxia, which can lead to alterations in genes associated with hypoxic phenotypes (6). OSA is closely associated with a genetic basis. Studies utilizing allele models have identified 10 genetic variants that are linked to an increased risk of OSA. These variants demonstrate odds ratios ranging from 1.21 to 2.07 in the global population, indicating a significant genetic contribution to the risk of developing OSA (7). Understanding these genetic changes not only provides insights into the mechanisms underlying OSA but may also pave the way for innovative and precise diagnostic methods.

Weighted gene co-expression network analysis (WGCNA) is a specialized statistical tool designed for an in-depth analysis of gene expression data (8). It identifies co-expression patterns among genes or transcripts, groups genes with similar expression traits, and pinpoints gene modules linked to specific biological processes or diseases. Unlike traditional methods, WGCNA uses a weighted network strategy to emphasize significant gene correlations, providing a systematic view of gene interactions and disease mechanisms. Recent research employing WGCNA reveals genetic factors for diseases, yet gaps remain in identifying markers for OSA and understanding their roles (9, 10). The goal of utilizing WGCNA in our study is to identify diagnostic genes associated with OSA (11, 12).

Mendelian randomization (MR) is a genetics-based approach designed to assess causal relationships between exposures and diseases. By leveraging genetic variants, such as SNPs, as instrumental variables, MR evaluates associations between environmental or lifestyle factors and disease risk. One of its inherent strengths is the random allocation of genes at conception, which ensures independence from many confounding factors, thereby facilitating a more unbiased assessment of causality (13, 14). To validate the core diagnostic genes associated with OSA, they were included in an MR analysis, building upon the genes previously identified through WGCNA. This study aims to enhance our understanding of the genetic foundations of OSA through the use of advanced bioinformatics tools like WGCNA and MR analysis. By identifying and validating genetic markers associated with OSA, we seek to develop more accurate diagnostic tools and targeted therapeutic strategies, ultimately reducing the substantial health and economic burdens of this disorder.

## Methods

### Differentially expressed genes of the OSA gene dataset

In R v4.1.2, the analysis began by loading the “limma” and “pheatmap” packages. Gene expression data were obtained from the GSE135917 dataset available in the GEO database (15). The dataset involved two distinct groups: a control group comprising 8 individuals and an OSA patient group consisting of 34 individuals. The diagnosis of OSA within this cohort was primarily reliant on the respiratory disturbance index (RDI). Following initial data processing, a differentially expressed gene (DEG) analysis was conducted using the “limma” package. A logFC threshold of 0.585, equivalent to a 1.5-fold change, and an adjusted *p*-value criterion of adjusted *p*-value <0.05 were applied to identify statistically significant genes. This threshold selection was based on common practices in other studies, ensuring that the identified gene changes were biologically meaningful and controlling the false positive rate, thereby ensuring the statistical validity and biological relevance of the results (16, 17). Subsequently, an expression heatmap was generated based on these identified genes.

### WGCNA analysis of gene expression

Utilizing the WGCNA approach, the normalized expression data were analyzed. Genes with fluctuations below 0.1 were excluded, and sample clustering was performed to eliminate outliers. An optimal soft threshold was determined based on the softPower criteria. Using the TOM algorithm and the specified softPower value, a gene adjacency matrix was constructed. Dynamic cutting was applied with a depth of 2 and a minimum module size of 100, and congruent modules were merged at a cut height of 0.35. Advanced analysis revealed correlations between modules and clinical markers, and core genes were identified by applying set thresholds: gene significance >0.5 and module association >0.8. These thresholds were chosen to ensure a high level of confidence in the biological significance of the findings, aligning with established practices in the field as illustrated in similar studies (18, 19).

### Diagnostic DEG identification and enrichment analysis

By intersecting datasets, potential diagnostic DEGs were identified. The “clusterProfiler” and “enrichplot” packages were then used to perform Kyoto Encyclopedia of Genes and Genomes (KEGG) and Gene Ontology (GO) enrichment analyses (12, 13). The GO analysis focused on three dimensions: biological process, cellular component, and molecular function. A *p*-value <0.05 was considered statistically significant.

### Diagnostic DEG analysis and interaction

The STRING database^1^ was used to evaluate diagnostic DEGs. Interactions among these genes were then visualized with Cytoscape v3.9.0. To refine the module’s density and significance within the protein–protein interaction networks, the “cytoHub” plug-in in Cytoscape was utilized.

### Receiver operating characteristic analysis for hub diagnostic genes diagnostic

The expression data from GSE13597 were meticulously analyzed using the “glmnet” and “pROC” packages. During this process, structured sample categorization was performed, hub diagnostic genes were identified and ranked, and receiver operating characteristic (ROC) curves were generated. These ROC curves provided an intuitive visual representation of the diagnostic capabilities of each core gene, with their performance quantitatively assessed using the area under the curve (AUC) values.

### Calibrating hub diagnostic gene model

The “rms” and “rmda” packages were used to extract gene expression data from the GSE135917 dataset and identify key diagnostic genes. Samples were categorized into “high” or “low” based on their expression profiles. To optimize the logistic regression modeling approach, the “datadist” function was employed for data structuring. The “lrm” function was then used to create a logistic regression model, with sample categories as dependent variables and gene expression classifications as predictors. From this model, a nomogram was generated using the “nomogram” function, providing a graphical representation of the “risk of disease” in relation to gene expression levels. Subsequently, a logistic regression analysis yielded a plotted nomogram and a calibration curve, confirming the model’s calibration accuracy.

### Immune cell infiltration and gene correlation analysis

The “CIBERSORT” method was utilized to evaluate immune cell infiltration within the expression data of the dataset (20). Cells were rigorously filtered based on a significance threshold of *p* < 0.05. For enhanced visualization, the “pheatmap” and “corrplot” packages were employed to generate heatmaps and correlation plots of immune cells, respectively. Subsequently, violin plots were constructed to provide a detailed depiction of the distribution of infiltrated immune cells. Furthermore, the relationship between the expression of the hub diagnostic gene and the abundance of immune cells was elucidated.

### MR analysis

An MR study was conducted using the “TwoSampleMR” package to explore potential causal relationships. Exposure data were obtained from various eQTL IDs, including eqtl-a-ENSG00000171848, eqtl-a-ENSG00000140307, eqtl-a-ENSG00000140650, eqtl-a-ENSG00000124802, and eqtl-a-ENSG00000153140. Outcome data were sourced from the ebi-a-GCST90018916 ID (21). Following extraction, datasets were harmonized, and appropriate instrumental variables for MR were identified. Subsequent MR analyses were performed, and results were converted to estimate odds ratios. Additionally, the heterogeneity and pleiotropy of the instrumental variables were critically assessed. To facilitate visual interpretation of the findings, scatter plots, forest plots, funnel plots, and leave-one-out sensitivity plots were generated, offering a comprehensive overview of the MR results and the robustness of the conclusions.

## Results

### Integrated analysis reveals the diagnostic genes

**Figure 1:**
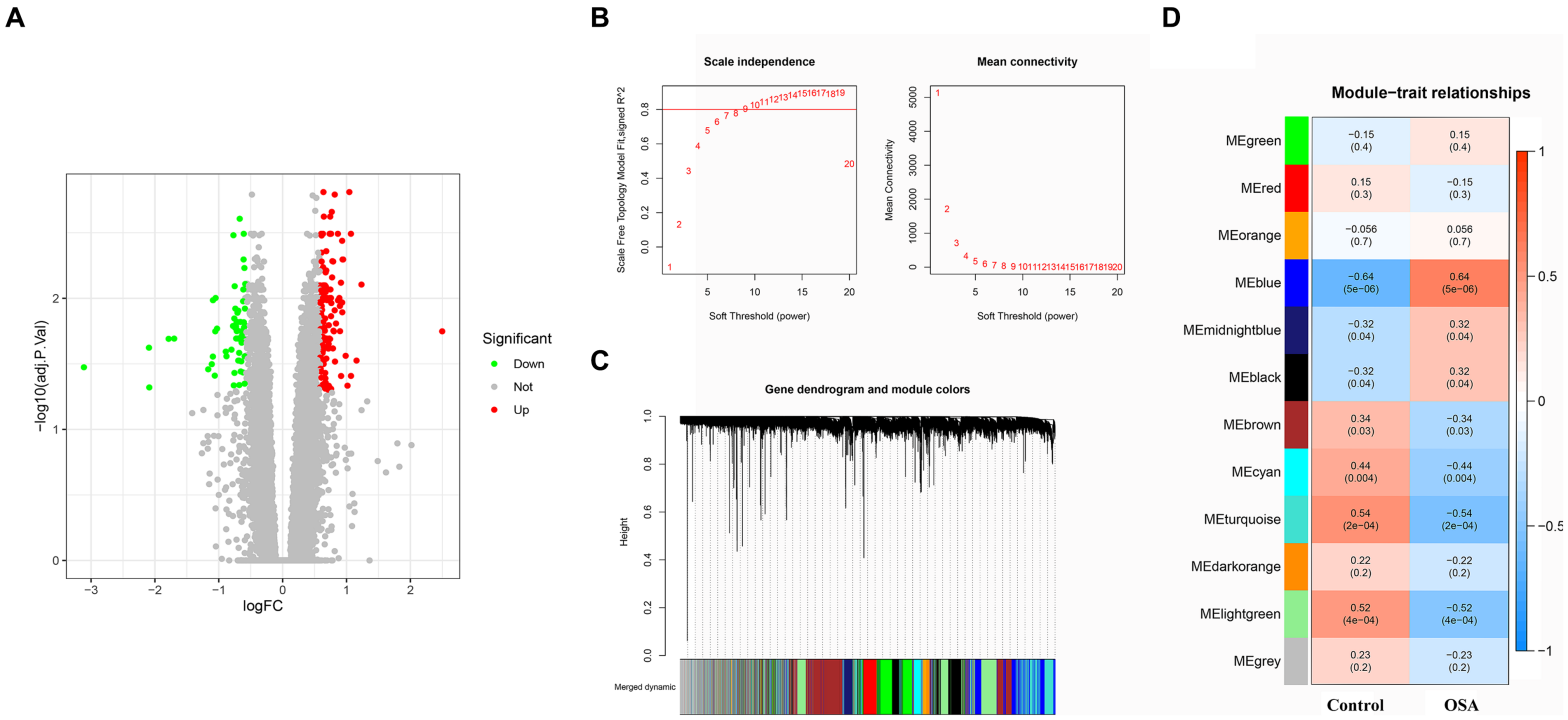
DEG and WGCNA analyses of OSA from the GSE135917 dataset were conducted. **(A)** Heatmap of 194 DEGs linked to OSA. **(B,C)** WGCNA analysis revealing the optimal soft-thresholding power at 12, ensuring scale-free topology in the gene co-expression network. **(D)** The “MEblue” module containing 2,502 DEGs strongly associated with OSA. DEGs, differentially expressed genes; WGCNA, weighted gene co-expression network analysis; OSA, obstructive sleep apnea.

A comprehensive DEG analysis was performed on the GSE135917 OSA gene dataset, resulting in the identification of 194 DEGs. These genes, which may play a pivotal role in the progression and manifestation of OSA, were visually represented in a heatmap (Figure 1A). To gain further insights into the interplay and co-expression patterns among these genes, WGCNA analysis was employed. This analysis identified an optimal soft threshold of 12, ensuring a scale-free topology in the gene network, as illustrated in Figures 1B,C. Within this network analysis, the “MEblue” module emerged as a significant player, demonstrating a strong association with OSA. This module alone comprises an extensive set of 2,502 DEGs (Figure 1D). The magnitude and co-expression patterns within this module underscore its critical significance in the development of OSA, suggesting it may harbor genes or pathways central to the molecular mechanisms of the disease.

### Diagnostic DEG enrichment analysis

**Figure 2:**
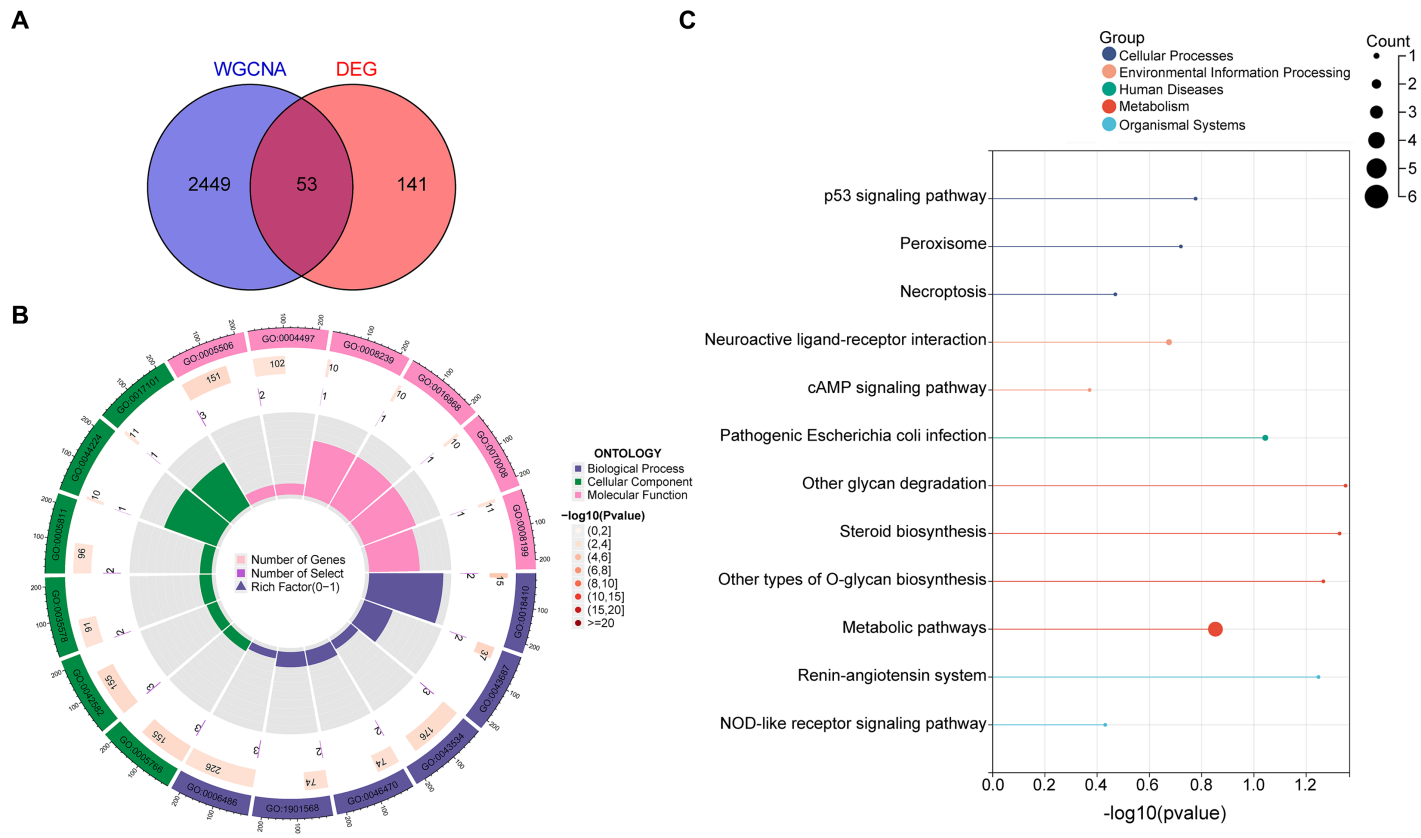
Diagnostic DEG enrichment analysis in OSA was conducted. **(A)** DEGs primarily associated with metabolic pathways. **(B)** GO enrichment analysis highlighting significant BPs, CCs, and MFs. **(C)** Principal pathways identified from KEGG analysis. DEGs, differentially expressed genes; GO, Gene Ontology; BP, biological process; CC, cellular component; MF, molecular function; KEGG, Kyoto Encyclopedia of Genes and Genomes.

By intersecting these datasets, 53 diagnostic DEGs predominantly associated with metabolic pathways were identified (Figure 2A). GO enrichment analysis highlighted key biological processes, including C-terminal protein amino acid modification, post-translational protein modification, blood vessel endothelial cell migration, and fatty acid derivative metabolic processes. In the cellular component category, the primary focus was on the lysosome and azurophil granule, while molecular function emphasized iron ion binding and monooxygenase activity (Figure 2B; Supplementary Figure S1). Additionally, KEGG analysis underscored metabolic pathways, the p53 signaling pathway, the cAMP signaling pathway, necroptosis, and peroxisome (Figure 2C).

### Hub diagnostic genes identification and modelling

**Figure 3:**
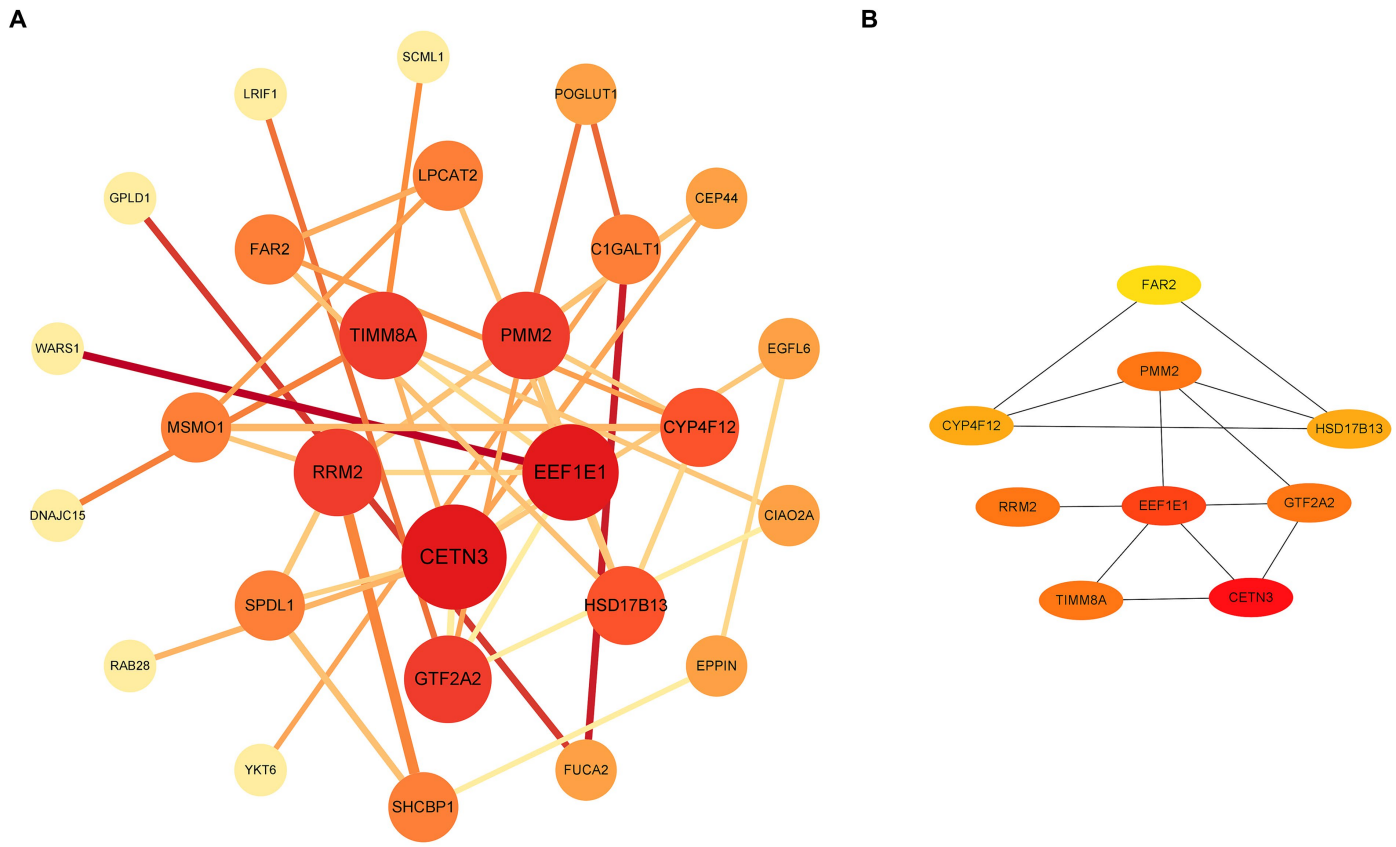
Analysis and visualization of diagnostic DEGs were performed. **(A)** Diagnostic DEG interactions mapped using the STRING database. **(B)** A highlighted representation of the hub diagnostic genes, including CETN3, EEF1E1, PMM2, GTF2A2, and RRM2, visualized with Cytoscape software. DEGs, differentially expressed genes.

**Figure 4:**
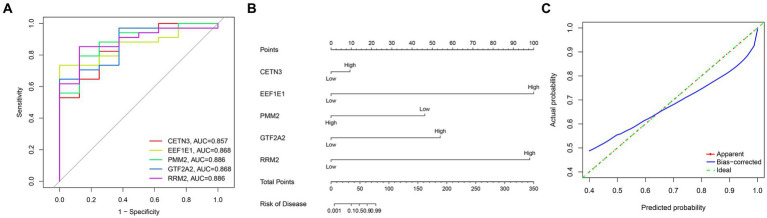
Comprehensive analysis of gene expression and disease risk assessment was conducted. **(A)** The diagnostic significance of the hub diagnostic genes. **(B)** A nomogram illustrating the direct correlation between gene expression levels and the associated “risk of disease.” **(C)** A model validation curve illustrating its accuracy.

Through a comprehensive exploration of the diagnostic DEGs using the STRING database and further visualization in Cytoscape software, essential core genes, including CETN3, EEF1E1, PMM2, GTF2A2, and RRM2, emerged prominently in their relevance (Figures 3A,B). Within the context of the GSE13597 dataset, these genes were identified as potential diagnostic cornerstones. Their diagnostic effectiveness was reinforced by an AUC value exceeding 0.85, demonstrating their strong diagnostic capability (Figure 4A).

Leveraging the predictive potential of key genes, including CETN3, EEF1E1, PMM2, GTF2A2, and RRM2, an advanced model was meticulously developed to assess disease susceptibility based on gene expression nuances. Using the nomogram function, a detailed nomogram was created, providing a clear visual representation of the direct association between gene expression levels and the “risk of disease” (Figure 4B). Following data collection and processing, a logistic regression was implemented. The resulting analysis produced a comprehensive nomogram that visually elucidates the probability of various outcomes, considering multiple predictors. To validate the accuracy and robustness of the established model, a calibration curve was generated. The remarkable alignment of this curve with the 45-degree reference line underscores the high consistency between predicted and observed outcomes, reaffirming the predictive strength of the model in categorizing gene expression levels (Figure 4C).

### Immune infiltration in patients with OSA

**Figure 5:**
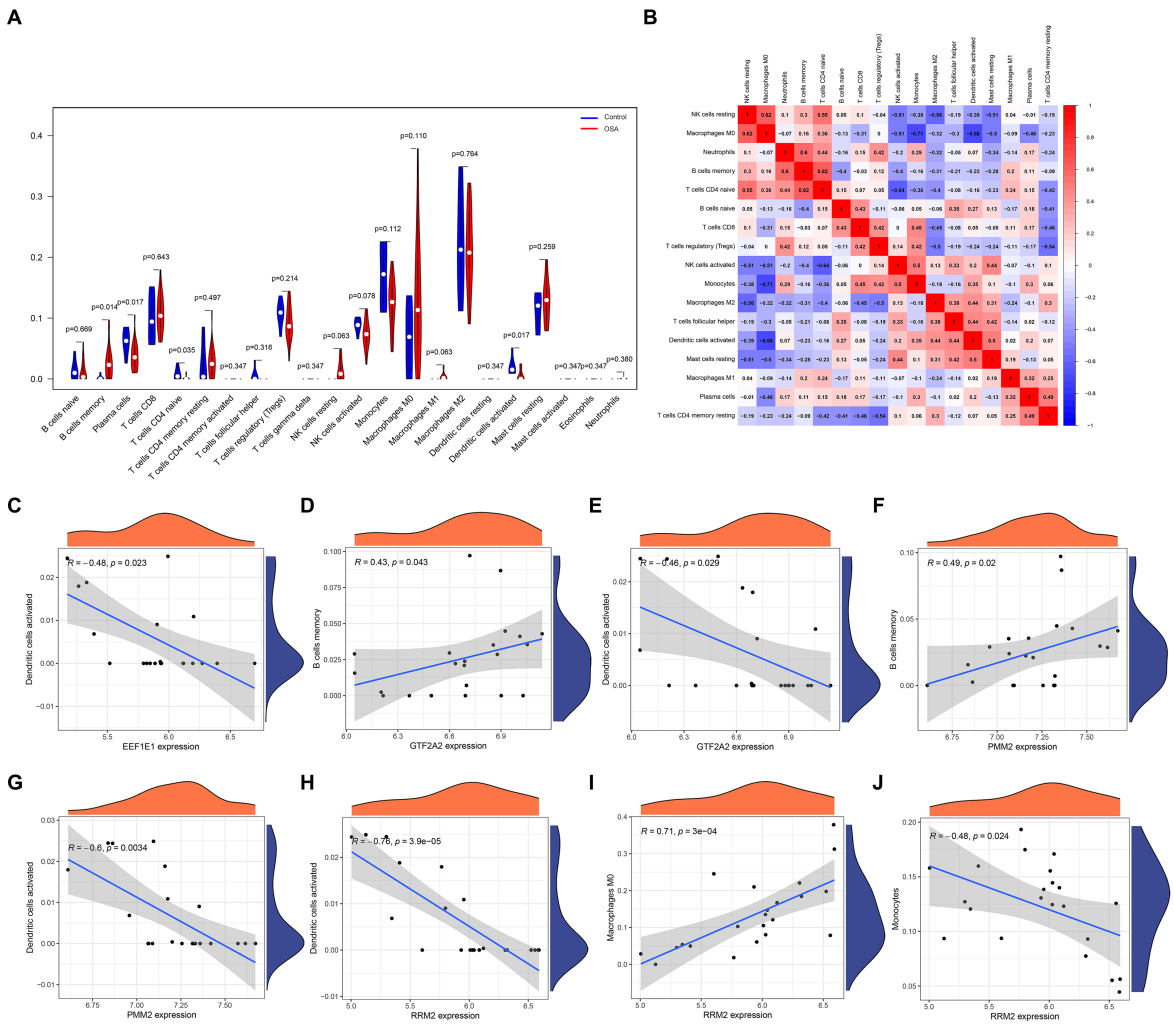
Immune infiltration analysis in patients with OSA and associations with the hub diagnostic genes was conducted. **(A)** Patterns in cellular abundance observed among patients with OSA. **(B)** Identified relationships and interactions among various immune cell types. **(C–J)** Graphic elucidations illustrating the intricate relationships between the hub diagnostic genes and immune cells.

In an in-depth analysis of immune infiltration within the dataset, specific patterns in cellular abundance were observed among patients with OSA. Notably, an increased presence of memory B cells and M0 macrophages suggested their potential role in OSA progression or response. Conversely, a noticeable decrease in plasma cells, naive T CD4 cells, and activated dendritic cells hinted at their diminished involvement in the OSA condition (Figures 5A,B). The intricate associations between these core genes and immune cells are visually illustrated in Figures 5C–J, elucidating the potential interactions and interplay between gene expression and immune cell profiles.

### MR analysis

**Figure 6:**
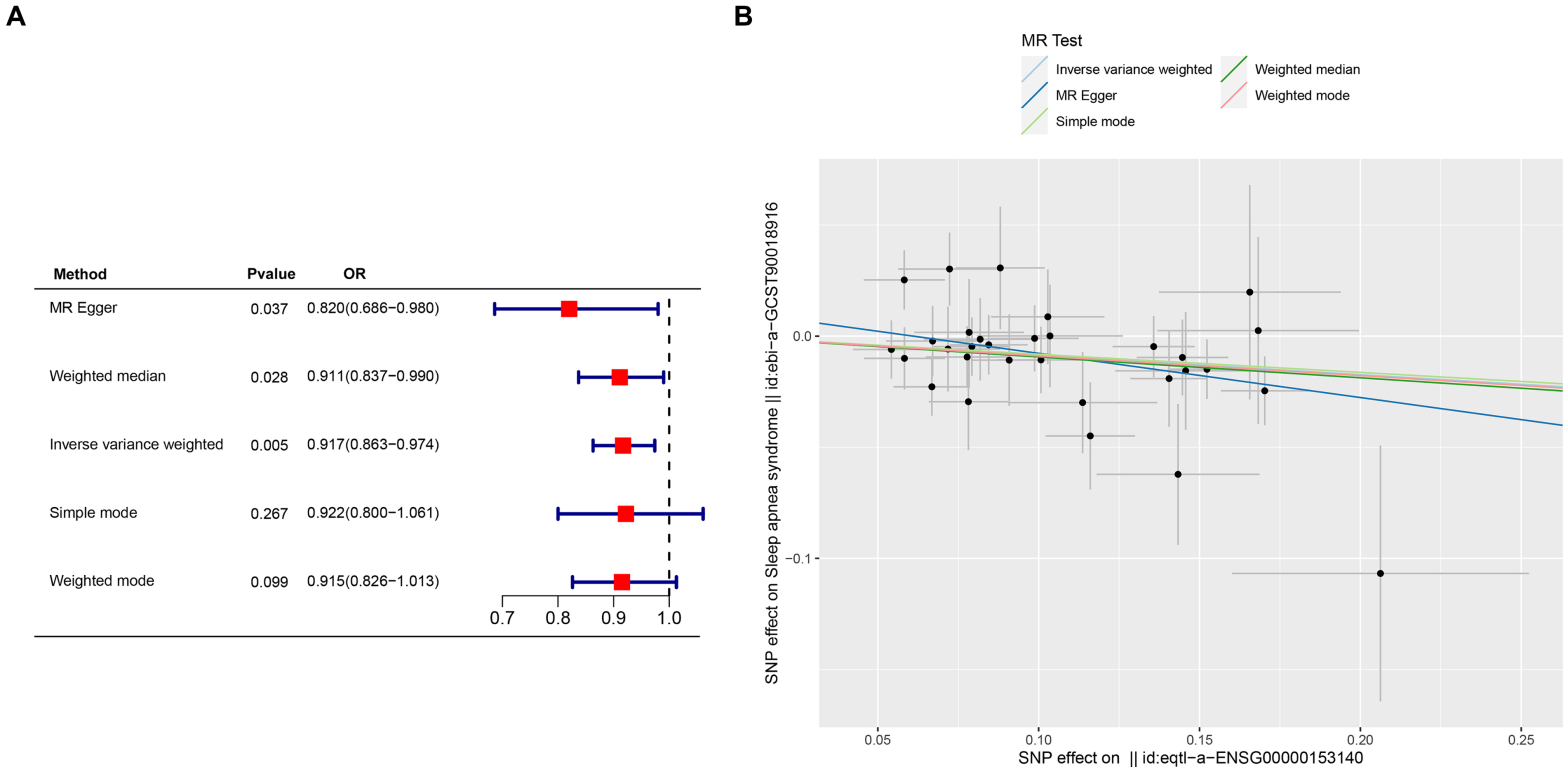
Mendelian randomization analysis was conducted to investigate gene-outcome associations. **(A)** Outcomes of the Mendelian randomization analysis presented in a forest plot, highlighting the significant association of CETN3. **(B)** Correlation between exposure and outcome depicted in a scatter plot, further emphasizing CETN3’s critical role.

**Figure 7:**
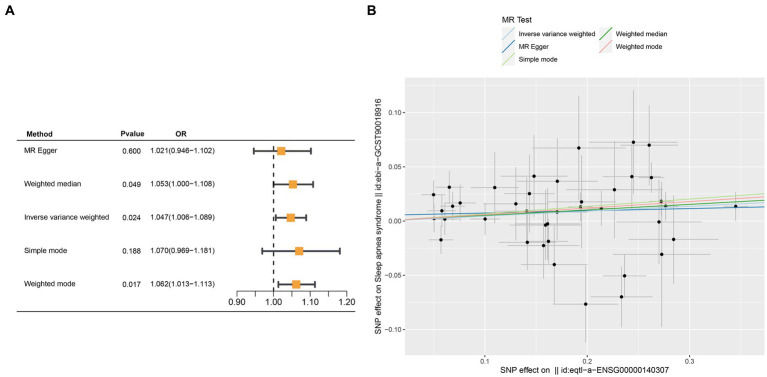
Mendelian randomization analysis was performed to examine GTF2A2-outcome associations. **(A)** Outcomes from the Mendelian randomization analysis presented in a forest plot, emphasizing the significant association of GTF2A2. **(B)** Correlation between exposure and outcome was detailed in a scatter plot, further spotlighting GTF2A2’s critical role.

Crucial insights into the potential causal relationships between certain genes and the designated outcome were provided by the MR analysis. CETN3, in particular, exhibited a clear and statistically significant association with the outcome, as indicated by the following *p*-values: IVW at 0.005, weighted median at 0.028, and MR Egger at 0.037. This compelling evidence suggests a noteworthy causal effect of CETN3 on the outcome (Figures 6A,B). GTF2A2 also emerged as a gene of significant interest, with its association with the outcome highlighted by the following *p*-values: 0.024 for IVW, 0.017 for weighted mode, and 0.026 for weighted median (Figures 7A–B).

Notably, the MR-Egger intercept method indicated no evidence of pleiotropy, and Cochran’s *Q* technique revealed an absence of heterogeneity (Table 1). These collective results provide compelling evidence of the nuanced associations between the genes CETN3 and GTF2A2 with the outcome, underscoring their significant linkage to OSA.

**Table 1:** Heterogeneity and horizontal pleiotropy analyses between CETN3, GTF2A2, and OSA.

Exposure	Outcome	Egger intercept	*p*-intercept	Cochran’s *Q*	*p*-value
CETN3	OSA	0.0121	0.199	24.027	0.728
GTF2A2	OSA	0.0053	0.460	52.804	0.156

## Discussion

OSA, a prevalent and severe sleep disorder, disrupts breathing during sleep. Typically, these interruptions last several seconds to a minute and occur when the throat muscles fail to keep the airway open, despite attempts to breathe. This obstruction often causes a decrease in blood oxygen levels and frequent awakenings throughout the night, leading to fragmented and non-restorative sleep (22, 23). The samples derived from GSE135917 originate from the subcutaneous adipose tissue of patients with OSA. This adipose tissue, readily accessible as a fat depot, plays a pivotal role in metabolic regulation.

The analysis included 8 healthy controls from Study Group 1 and 34 patients with OSA from both groups. The GSE135917 OSA gene dataset was thoroughly examined, leading to the identification of 194 DEGs crucial to the progression and manifestation of OSA. Network analysis via WGCNA identified a strong association of the “MEblue” module with OSA, indicating that this module may contain key genes or pathways central to the disease’s molecular mechanisms. WGCNA has become a prominent tool in OSA research due to its ability to systematically explore the molecular complexities of the disorder (24, 25). Utilizing WGCNA, gene co-expression modules relevant to OSA can be identified, critical driver genes can be discovered, and various data types such as transcriptomics, proteomics, and metabolomics can be integrated, providing a comprehensive understanding of the disease landscape (26, 27).

Through a thorough analysis of datasets, 53 diagnostic DEGs predominantly associated with metabolic pathways were precisely identified. These DEGs provide profound insights into the potential molecular mechanisms underlying OSA, particularly in terms of metabolic regulation. Identified biological processes, such as C-terminal protein amino acid modification, post-translational protein modification, blood vessel endothelial cell migration, and fatty acid derivative metabolic processes, are intriguing. These processes could potentially be linked to metabolic abnormalities, vascular dysfunction, and delayed tissue repair often observed in patients with OSA (28). Regarding cellular components, the prominence of the primary lysosome and azurophil granule suggests an impact of OSA on cellular acidic environments and inflammatory responses. Notably, azurophil granules are associated with inflammatory responses in various ailments (29). Insights into molecular function, highlighting iron ion binding and monooxygenase activity, suggest a potential connection between OSA and red cell functionality and the oxidative stress response at the tissue level (30). The STRING database, combined with Cytoscape software, facilitated a detailed examination of diagnostic DEGs, uncovering the intricate interrelationships among these genes (31). Subsequent ROC curve analyses emphasized the robust diagnostic potential of the identified core genes. Additionally, the established gene model, utilizing logistic regression and model calibration, offers profound insights into the disease and illuminates new avenues for future diagnostic and therapeutic interventions.

The unique dynamics of immune cells in patients with OSA were revealed through an in-depth analysis of immune infiltration. Memory B cells and M0 macrophages showed a significant increase, suggesting their role in mediating inflammatory cascades and subsequent tissue impairments associated with the disease (32). In contrast, plasma cells, naive T CD4 cells, and activated dendritic cells exhibited a reduced prevalence, indicating a diminished regulatory capacity as the disease progresses, likely linked to the chronic hypoxic environment and persistent inflammation inherent in OSA (33). Particularly, the reduction in plasma cells may lead to weakened antibody-mediated immune responses in patients with OSA, diminishing their defense against pathogens (34). The decrease in naive T CD4 cells could affect the regulatory and activation functions of the immune system in OSA patients, weakening their resistance to infections (35). Furthermore, a reduction in activated dendritic cells suggests that OSA may interfere with effective antigen presentation and the initiation of immune responses, impacting overall immune regulation. These insights, combined with the complex interplay between cellular components and key genes, pave the way for a deeper exploration of the molecular and immunological foundations of OSA. Several studies have delved into SNPs and their connection to OSA, highlighting the discovery of rs11691765 in GPR83 and rs35424364 in C6ORF183 within the Hispanic/Latino American population. These genomic-level findings shed new light on the roles of inflammation and hypoxia signaling pathways in sleep apnea (36). OSA demonstrates distinct genetic disparities among various ethnic groups. In the case of European Americans, genetic variants in CRP and GDNF show a significant association with the AHI. Conversely, in African Americans, the rs9526240 variant within the HTR2A gene is notably correlated with the presence of OSA (37). Our study distinctively emphasized MR analysis within a European population, providing specialized insight into genetic influences. While our findings were primarily based on this demographic, we acknowledged the importance of comparing these results with SNP data reported in Hispanic/Latino and African American populations to understand broader genetic implications. The MR approach revealed pronounced correlations between certain genes and outcomes relevant to OSA. CETN3, in particular, showed distinct associations across various methodologies, underscoring its critical role in the genetic framework of OSA. Similarly, GTF2A2 emerged as another significant contributor within the genetic context of the disease. On the other hand, several genes, including EEF1E1, PMM2, and RRM2, did not exhibit robust associations, highlighting the nuanced and multifaceted genetic architecture of OSA.

Investigations into family genetics have shown that in OSA, inherited traits may influence late sleep timing associated with increased IL-6 levels, and a genetic tendency towards more significant social jetlag corresponding with higher IL-1 levels (38). The genetic relationship between OSA and its pathological features is evident, as demonstrated by a twin study from Hungary on OSA. Specifically, the study found a significant shared genetic basis linking serum triglyceride levels with key indicators of OSA severity, such as the oxygen desaturation index and the proportion of sleep time with oxygen saturation below 90% (39). In summary, it is evident that genetic variations significantly contribute to the development and progression of OSA, underlining the importance of genetic factors in understanding and addressing this condition. CETN3, also known as centrin 3, encodes a protein belonging to the EF-hand protein superfamily. As calcium-binding proteins, centrins play a crucial role in centrosome dynamics, particularly in centrosome replication and separation, both essential for cell division (40, 41). Oxidative stress, commonly associated with conditions like OSA, can disrupt the cell cycle by affecting both protein functions and DNA integrity (42). Elevated oxidative stress could, therefore, compromise the functional integrity of CETN3, hindering its primary role in maintaining centrosome dynamics. Studies have shown that disruptions in cell cycle regulation are linked to sleep disturbances and respiratory dysfunction, highlighting the relevance of CETN3 in OSA pathology (43). Similarly, GTF2A2 encodes a critical subunit of the general transcription factor IIA, which is essential for the assembly of the preinitiation complex in gene transcription directed by RNA polymerase II. Composed of two main subunits, GTF2A2 represents one of them (44, 45). Given GTF2A2’s central role in transcription initiation, oxidative stress induced by elevated ROS levels, often seen in OSA, might impede its function or expression (46). The potential of ROS to alter transcription regulators and their target genes suggests that the cellular imbalances caused by OSA could indirectly modulate the function of genes such as GTF2A2. This impairment may lead to altered transcriptional regulation, which has been shown to affect cellular function and contribute to the systemic effects observed in OSA patients, such as enhanced inflammatory responses and metabolic dysregulation (47).

Additionally, we recognize that due to limited sample sizes and selection biases, our findings may need to be validated in a broader population to confirm their generalizability. Variations and potential biases may occur from exclusive reliance on specific datasets and sample origins. Despite strong associations identified with CETN3 and GTF2A2, further investigation is necessary for genes such as EEF1E1, PMM2, and RRM2. It’s critical to experimentally validate the causal roles of these genes in OSA. Although the MR approach is robust, it requires cautious interpretation due to its foundational assumptions. Future research should expand to include a wider range of tissues and functional validations to deepen our understanding.

## Conclusion

In-depth analysis has identified critical genes, notably CETN3 and GTF2A2, with potential roles in the etiology and progression of OSA. Insights into immune cell dynamics further emphasize the multifaceted nature of the disease. While promising, inherent limitations in the study must be considered, particularly concerning potential biases in the datasets and assumptions in the methodology. These findings offer a foundation for future OSA research, highlighting the need for experimental validation and broader exploration.

## Data Availability

The original contributions presented in the study are included in the article/Supplementary material, further inquiries can be directed to the corresponding author.

## Footnotes

^1^ https://cn.string-db.org/
