# Supplementary figures and images for: Identification and Mendelian randomization validation of pathogenic gene biomarkers in obstructive sleep apnea
Source: Front Neurol. 2024 Aug 16;15:1442835. doi: 10.3389/fneur.2024.1442835 (PMC11363542; doi:10.3389/fneur.2024.1442835)

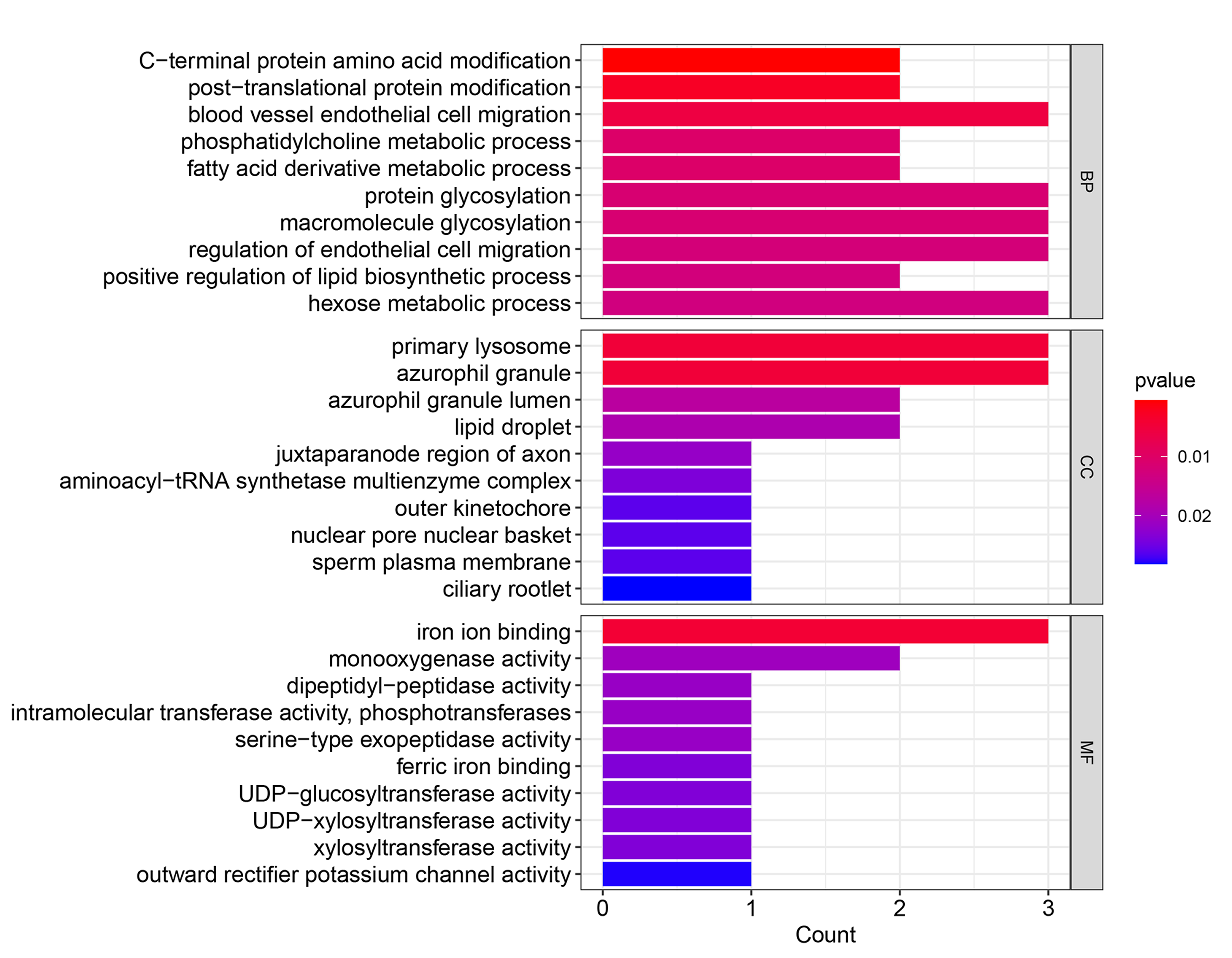

Supplement: Supplementary file 1 [file Image_1.TIF]
