# Supplementary material for: Identification and Mendelian randomization validation of pathogenic gene biomarkers in obstructive sleep apnea
Source: Front Neurol. 2024 Aug 16;15:1442835. doi: 10.3389/fneur.2024.1442835 (PMC11363542; doi:10.3389/fneur.2024.1442835)
Supplement: Supplementary file 2 [file Table_1.DOCX]

**Table S1| Data from the subject diagnosed with OSA and from the healthy individual.**

| Group | Source name | Age | Sex | Bmi |
| --- | --- | --- | --- | --- |
| Normal Subject 1 | Subcutaneous Fat Tissue | 66 | F | 31.3 |
| Normal Subject 2 | Subcutaneous Fat Tissue | 53 | F | 35.2 |
| Normal Subject 3 | Subcutaneous Fat Tissue | 61 | F | 41.7 |
| Normal Subject 4 | Subcutaneous Fat Tissue | 30 | F | 40.3 |
| Normal Subject 5 | Subcutaneous Fat Tissue | 56 | M | 42.5 |
| Normal Subject 6 | Subcutaneous Fat Tissue | 47 | F | 26.7 |
| Normal Subject 7 | Subcutaneous Fat Tissue | 60 | F | 33.6 |
| Normal Subject 8 | Subcutaneous Fat Tissue | 63 | F | 30.4 |
| OSA Subject 1 | Subcutaneous Fat Tissue | 34 | F | 50.9 |
| OSA Subject 2 | Subcutaneous Fat Tissue | 52 | F | 28.8 |
| OSA Subject 3 | Subcutaneous Fat Tissue | 64 | F | 32.4 |
| OSA Subject 4 | Subcutaneous Fat Tissue | 55 | F | 32.7 |
| OSA Subject 5 | Subcutaneous Fat Tissue | 58 | M | 35.5 |
| OSA Subject 6 | Subcutaneous Fat Tissue | 70 | F | 39.1 |
| OSA Subject 7 | Subcutaneous Fat Tissue | 62 | F | 20.6 |
| OSA Subject 8 | Subcutaneous Fat Tissue | 50 | F | 39.1 |
| OSA Subject 9 | Subcutaneous Fat Tissue | 48 | M | 50.3 |
| OSA Subject 10 | Subcutaneous Fat Tissue | 68 | M | 32 |
| OSA Subject 11 | Subcutaneous Fat Tissue | 69 | M | 32.2 |
| OSA Subject 12 | Subcutaneous Fat Tissue | 34 | M | 37.5 |
| OSA Subject 13 | Subcutaneous Fat Tissue | 54 | M | 32.2 |
| OSA Subject 14 | Subcutaneous Fat Tissue | 51 | F | 43.1 |
| OSA Subject 15 | Subcutaneous Fat Tissue | 34 | F | 59.4 |
| OSA Subject 16 | Subcutaneous Fat Tissue | 67 | M | 39.8 |
| OSA Subject 17 | Subcutaneous Fat Tissue | 52 | F | 37.8 |
| OSA Subject 18 | Subcutaneous Fat Tissue | 58 | F | 36.4 |
| OSA Subject 19 | Subcutaneous Fat Tissue | 54 | M | 61 |
| OSA Subject 20 | Subcutaneous Fat Tissue | 48 | F | 41.2 |
| OSA Subject 21 | Subcutaneous Fat Tissue | 59 | M | 30.3 |
| OSA Subject 22 | Subcutaneous Fat Tissue | 57 | F | 39.1 |
| OSA Subject 23 | Subcutaneous Fat Tissue | 47 | M | 62.5 |
| OSA Subject 24 | Subcutaneous Fat Tissue | 42 | M | 37.5 |
| OSA Subject 25 | Subcutaneous Fat Tissue | 68 | F | 49.2 |
| OSA Subject 26 | Subcutaneous Fat Tissue | 43 | F | 29.3 |
| OSA Subject 27 | Subcutaneous Fat Tissue | 32 | F | 54.1 |
| OSA Subject 28 | Subcutaneous Fat Tissue | 35 | M | 40.2 |
| OSA Subject 29 | Subcutaneous Fat Tissue | 34 | F | 53.8 |
| OSA Subject 30 | Subcutaneous Fat Tissue | 51 | M | 37.4 |
| OSA Subject 31 | Subcutaneous Fat Tissue | 54 | M | 37.8 |
| OSA Subject 32 | Subcutaneous Fat Tissue | 53 | M | 43.5 |
| OSA Subject 33 | Subcutaneous Fat Tissue | 46 | F | 44 |
| OSA Subject 34 | Subcutaneous Fat Tissue | 46 | M | 42.3 |

**Annotations:**

BMI: Body Mass Index; Age: The age of the subject in years; Sex: The gender of the subject (F: Female, M: Male);

Group: The category to which the subject belongs (Normal or OSA).
